# Supplementary material for: Evolutionary discovery and characterization of fungal transcriptional activators using active learning
Source: bioRxiv. 2025 Oct 29:2025.09.12.675635. Preprint. [Version 2] doi: 10.1101/2025.09.12.675635 (PMC12636428; doi:10.1101/2025.09.12.675635)
Supplement: Supplement 6 [file NIHPP2025.09.12.675635v2-supplement-6.pdf]

bioRxiv preprint doi: https://doi.org/10.1101/2025.09.12.675635; this version posted October 29, 2025. The copyright holder for this preprint (which was not certified by peer review) is the author/funder, who has granted bioRxiv a license to display the preprint in perpetuity. It is made available under aCC-BY 4.0 International license.

Supplementary Figures

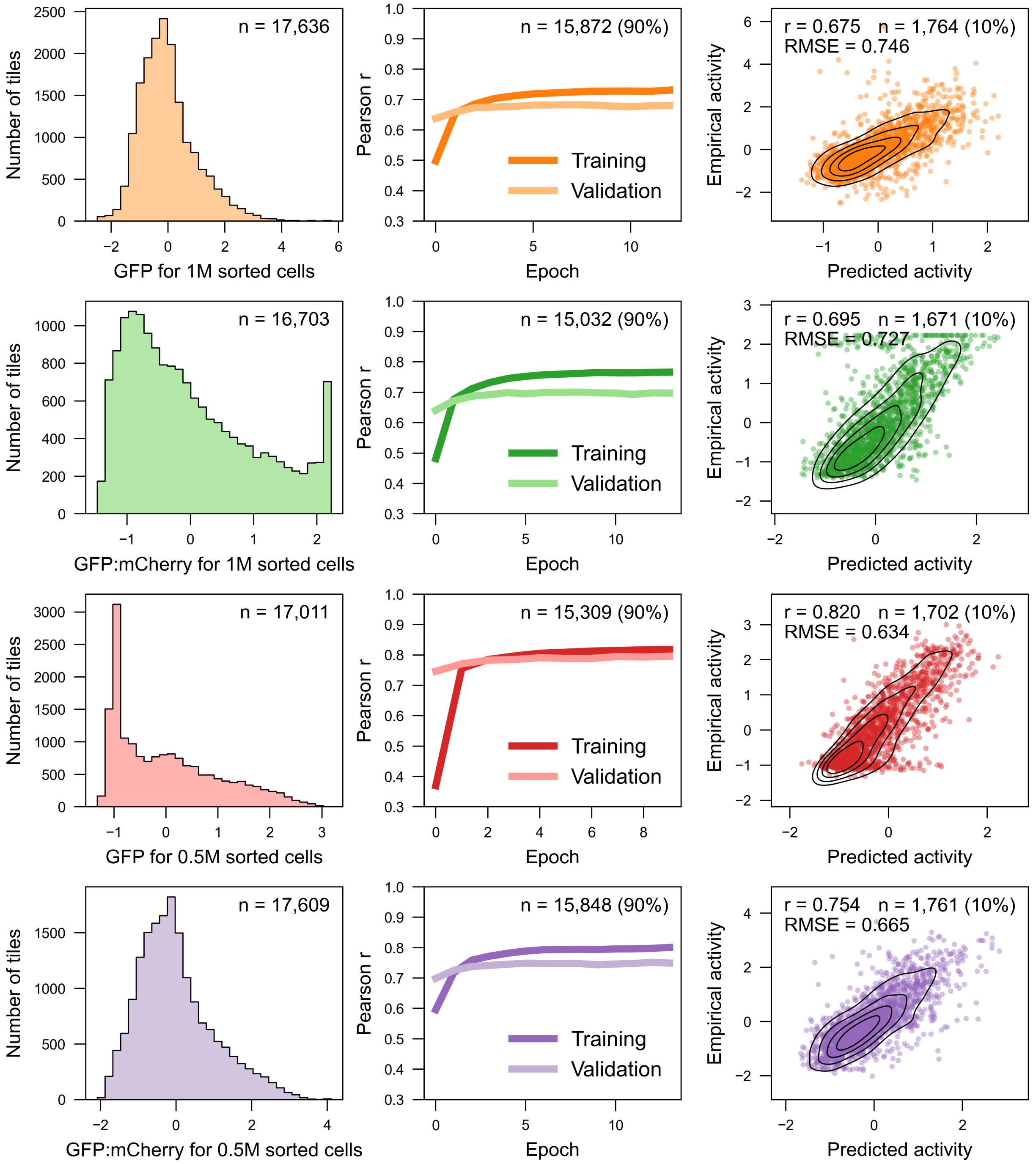

Figure S1. Selection of AD activity metric. The AD activity measurement was selected based on the distribution of empirical activity. We used the GFP:mCherry ratio for 500,000 sorted cells for sequence-to-function modeling of transcriptional activators.

bioRxiv preprint doi: https://doi.org/10.1101/2025.09.12.675635; this version posted October 29, 2025. The copyright holder for this preprint (which was not certified by peer review) is the author/funder, who has granted bioRxiv a license to display the preprint in perpetuity. It is made available under aCC-BY 4.0 International license.

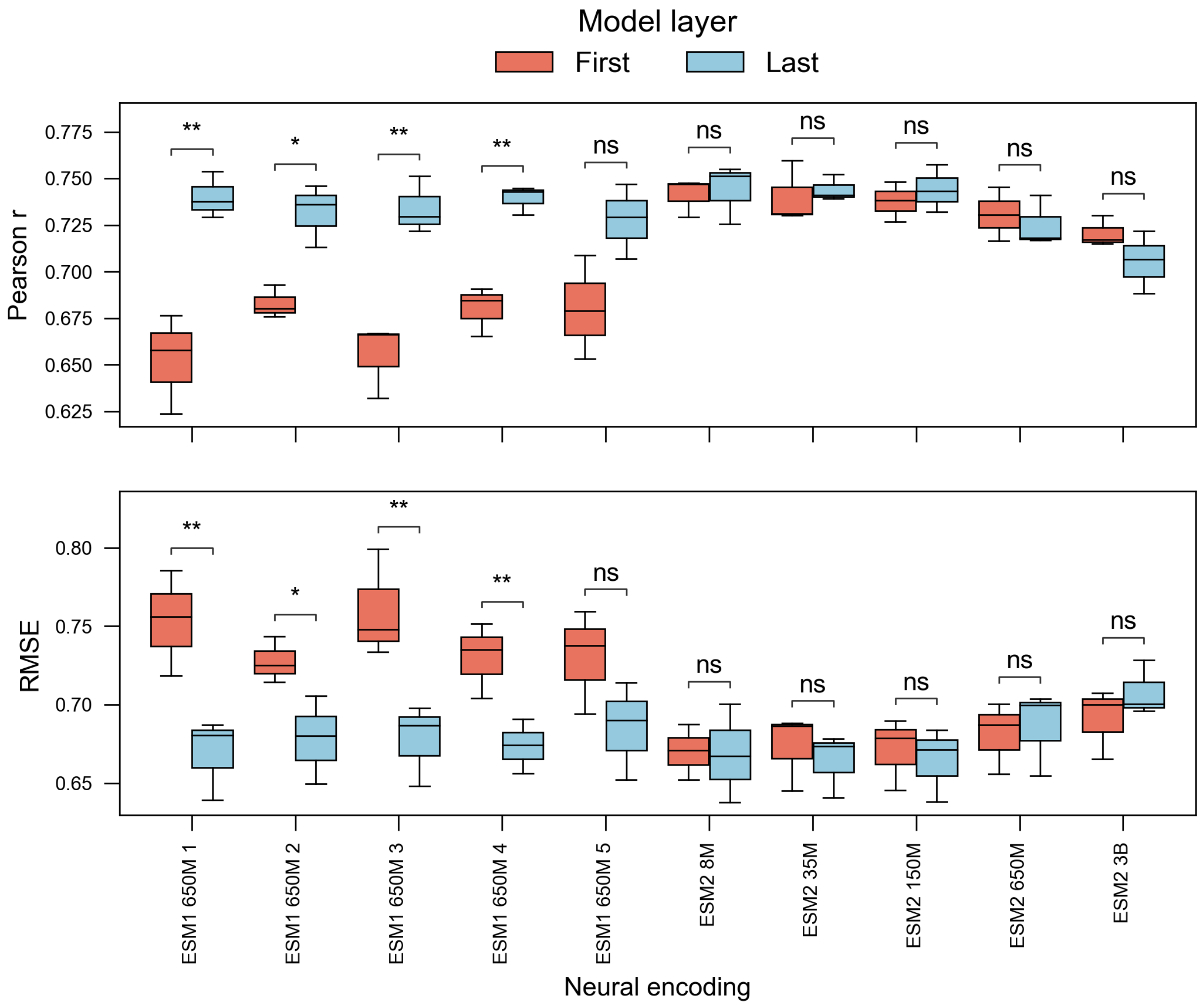

Figure S2. Selection of protein sequence encoding. Neural encodings slightly outperform simple encodings and were selected for predictive modeling. A two-sided Student's t-test with Bonferroni multiple comparison correction was used to assess statistical significance between the first and last layer of each ESM model. Results show that encodings from the first layer versus the last layer of version 1 models have statistically significant differences in performance on a held-out test dataset (ns: 5e-2 < p ≤ 1; *: 1e-2 < p ≤ 5e-2; **: 1e-3 < p ≤ 1e-2; ***: 1e-4 < p ≤ 1e-3, Student's t-test with Bonferroni multiple comparison correction). Each model and layer combination was evaluated across 3 random seeds.

bioRxiv preprint doi: https://doi.org/10.1101/2025.09.12.675635; this version posted October 29, 2025. The copyright holder for this preprint (which was not certified by peer review) is the author/funder, who has granted bioRxiv a license to display the preprint in perpetuity. It is made available under aCC-BY 4.0 International license.

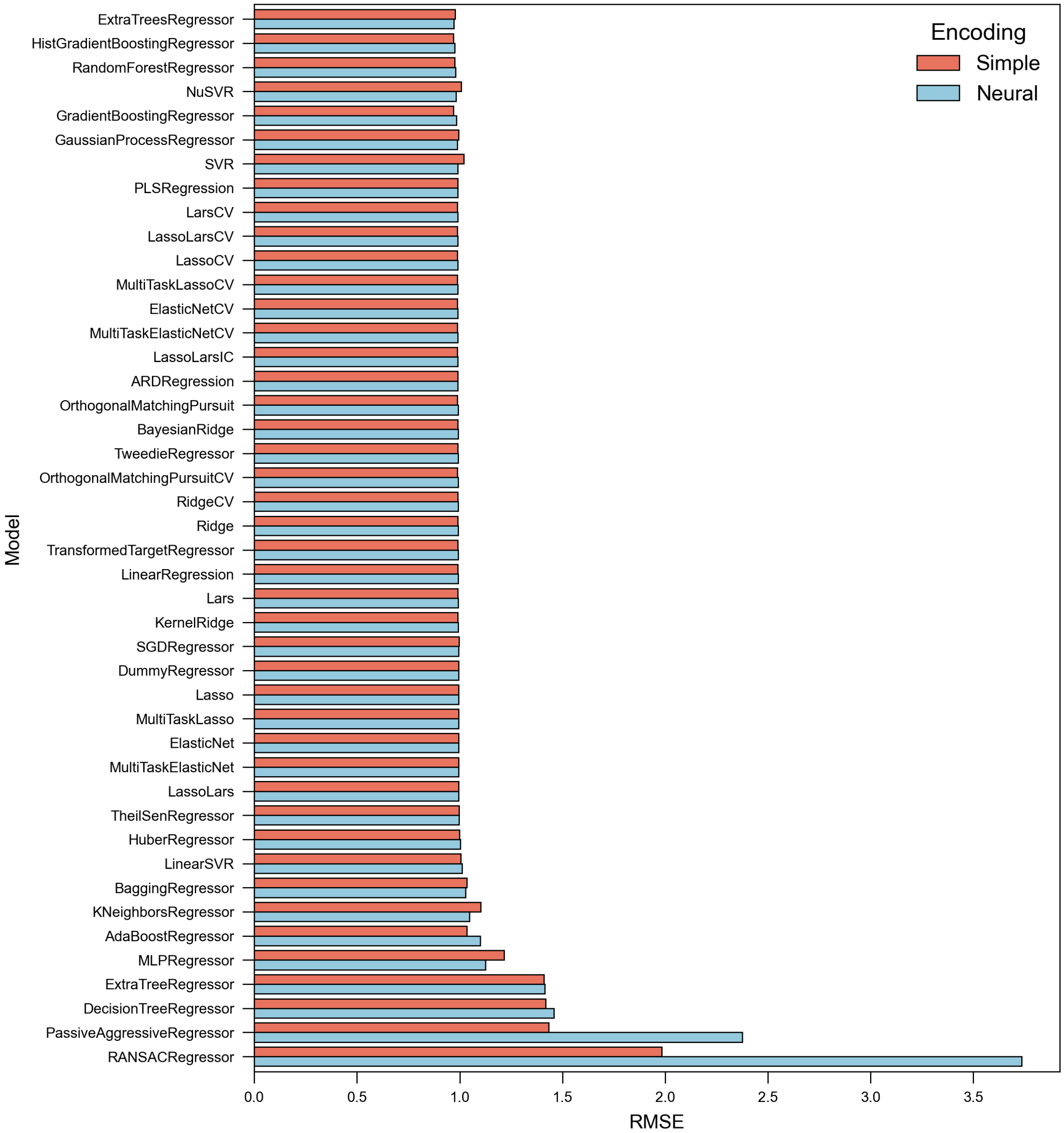

606 **Figure S3. Evaluation of lightweight regressors.** We evaluated 44 lightweight regressors trained with
607 simple integer encodings or pretrained neural encodings. All models performed poorly on the held-out test
608 dataset, leading us to consider more complex architectures for quantifying transcriptional activators.

bioRxiv preprint doi: https://doi.org/10.1101/2025.09.12.675635; this version posted October 29, 2025. The copyright holder for this preprint (which was not certified by peer review) is the author/funder, who has granted bioRxiv a license to display the preprint in perpetuity. It is made available under aCC-BY 4.0 International license.

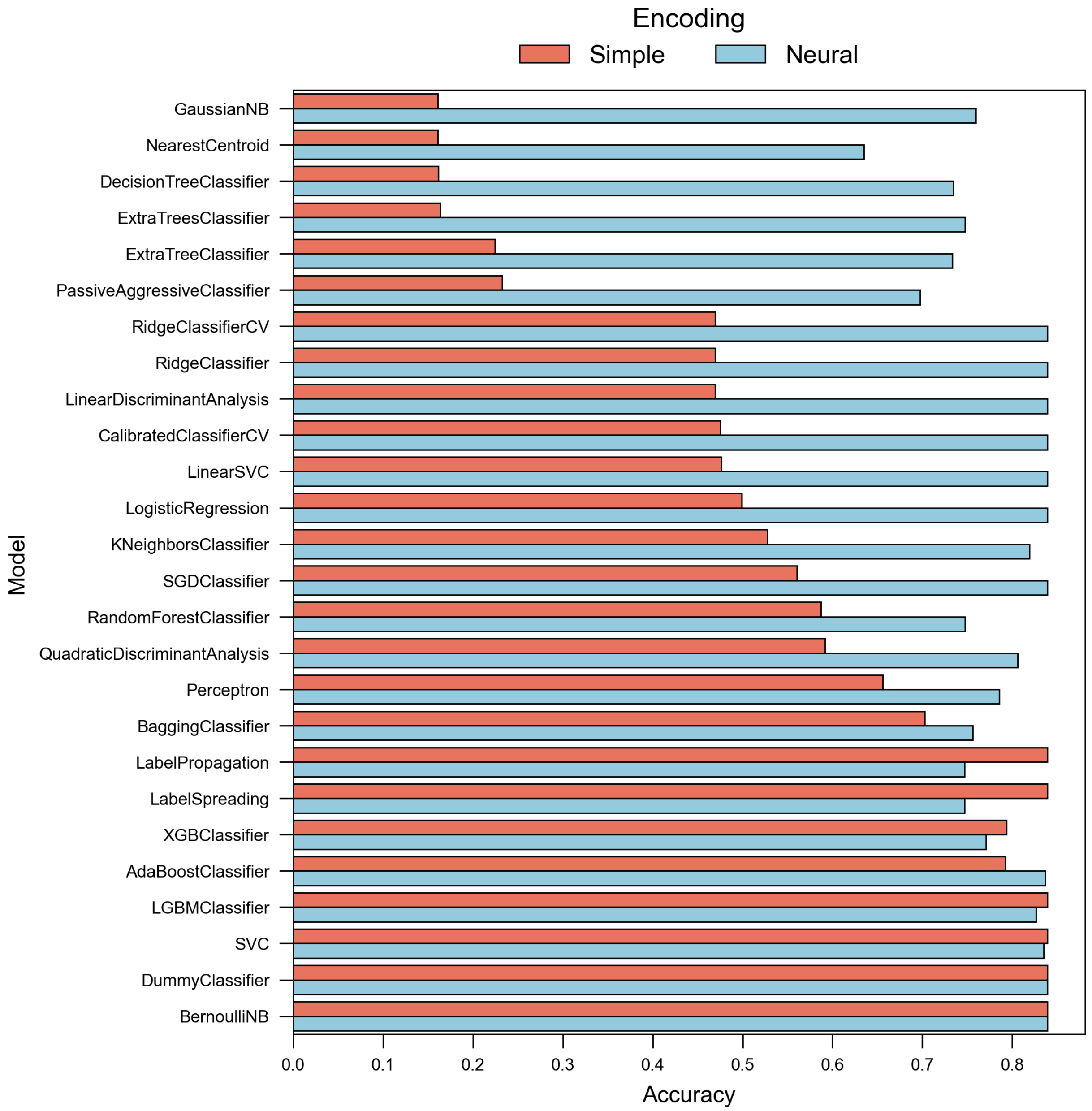

Figure S4. Evaluation of lightweight classifiers. Despite poor performance of the lightweight regressors, several lightweight classifiers had high accuracy performed on the binary classification task. These results indicate that the classification task has lower complexity than the regression task and that several classifiers perform better when trained with neural encodings over simple encodings.

bioRxiv preprint doi: https://doi.org/10.1101/2025.09.12.675635; this version posted October 29, 2025. The copyright holder for this preprint (which was not certified by peer review) is the author/funder, who has granted bioRxiv a license to display the preprint in perpetuity. It is made available under aCC-BY 4.0 International license.

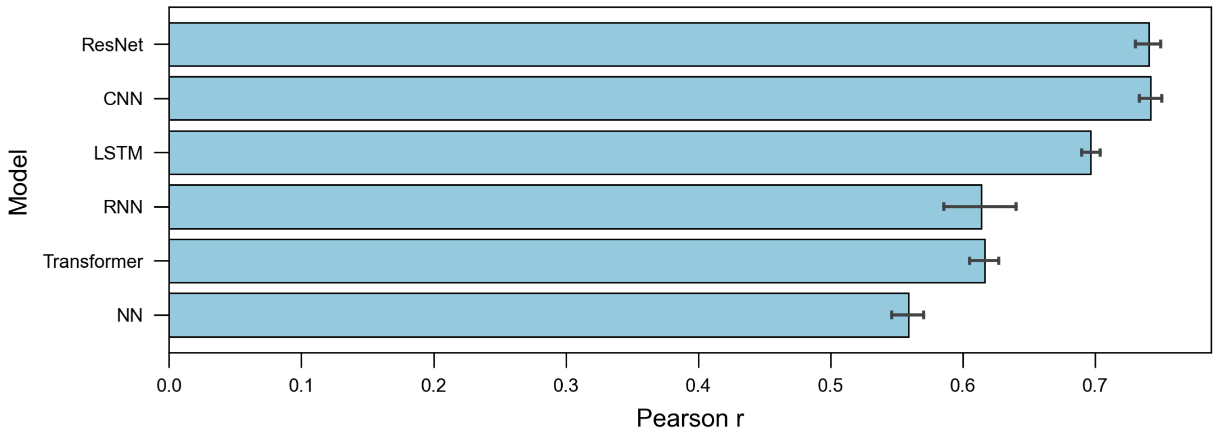

Figure S5. Selection of surrogate model architecture. Complex architectures outperform lightweight regressors on a held-out test dataset. Models incorporating convolutional layers, specifically a CNN and a CNN with residual connections (ResNet), achieve the highest prediction performance. Each model was evaluated across 10 random seeds.

bioRxiv preprint doi: https://doi.org/10.1101/2025.09.12.675635; this version posted October 29, 2025. The copyright holder for this preprint (which was not certified by peer review) is the author/funder, who has granted bioRxiv a license to display the preprint in perpetuity. It is made available under aCC-BY 4.0 International license.

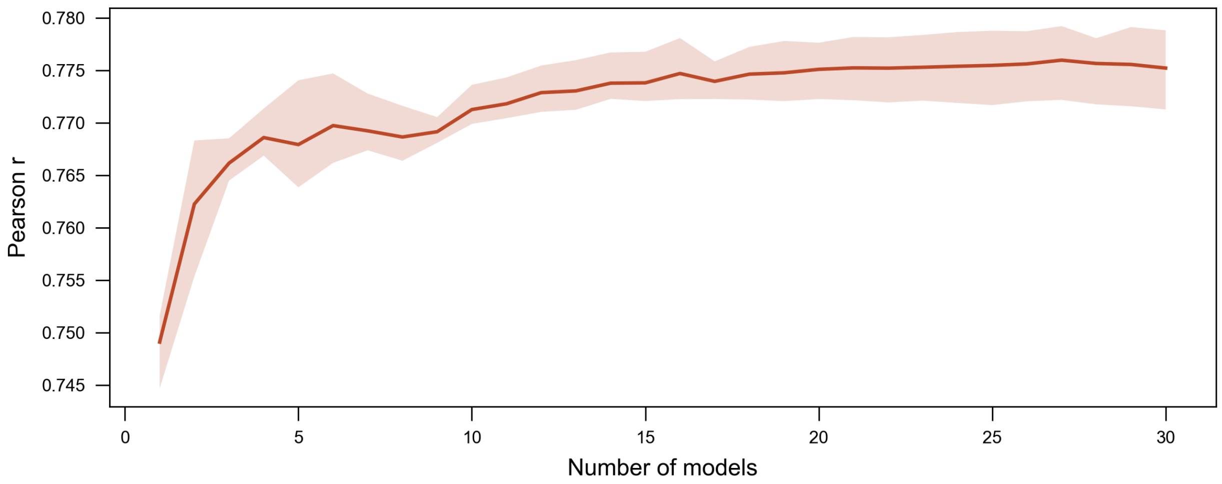

Figure S6. Deep ensembling of ADhunter. Improvement in ensemble model performance with respect to ensemble size on a held-out test dataset across 10 random seeds.

bioRxiv preprint doi: https://doi.org/10.1101/2025.09.12.675635; this version posted October 29, 2025. The copyright holder for this preprint (which was not certified by peer review) is the author/funder, who has granted bioRxiv a license to display the preprint in perpetuity. It is made available under aCC-BY 4.0 International license.

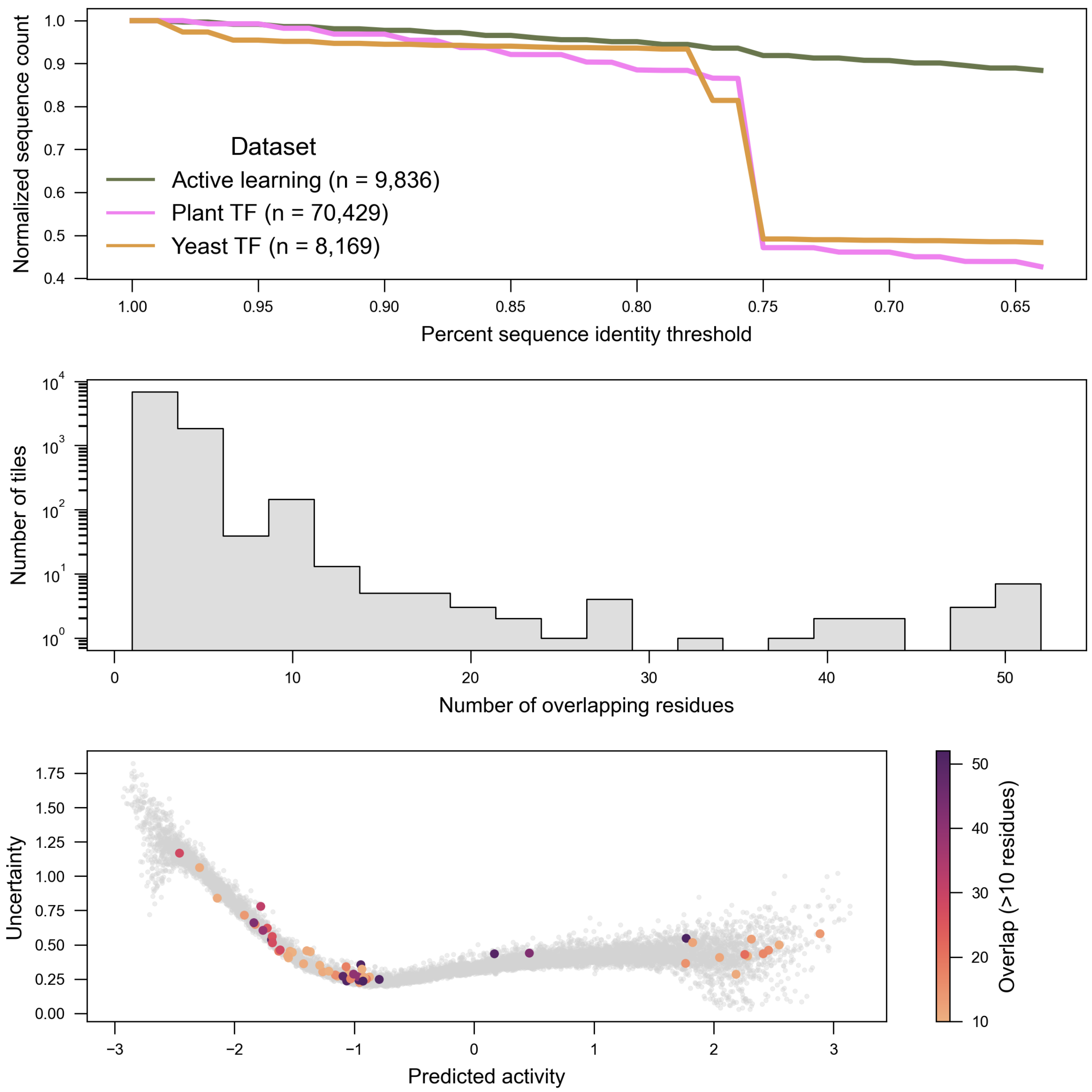

619 **Figure S7. Dataset sequence identity clustering.** Each dataset was clustered by sequence identity and the active learning dataset selected from the MycoCosm collection is shown to contain the most diversity. Within the test dataset, the median sequence overlap between tiles is 3 residues, where tiles with more than 10 overlapping residues are selected from across the range of predicted activity.

bioRxiv preprint doi: https://doi.org/10.1101/2025.09.12.675635; this version posted October 29, 2025. The copyright holder for this preprint (which was not certified by peer review) is the author/funder, who has granted bioRxiv a license to display the preprint in perpetuity. It is made available under aCC-BY 4.0 International license.

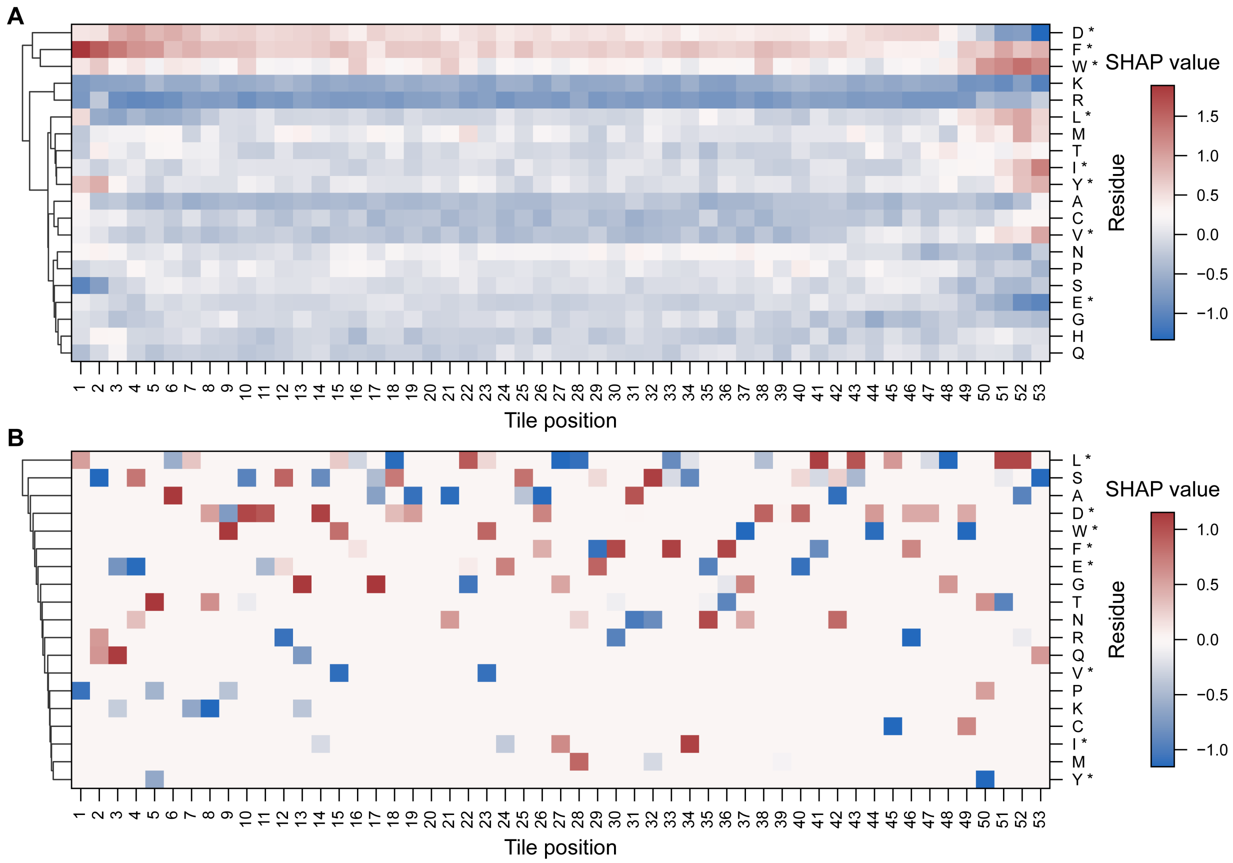

Figure S8. Model interpretability analysis of transcriptional activators. (A) SHAP analysis shows the majority of functional determinants are within the first and last 8 residues. (B) Strong activators (i.e., mean + 1 s.d. as defined in Morffy et al.) have very specific sequence grammar.

bioRxiv preprint doi: https://doi.org/10.1101/2025.09.12.675635; this version posted October 29, 2025. The copyright holder for this preprint (which was not certified by peer review) is the author/funder, who has granted bioRxiv a license to display the preprint in perpetuity. It is made available under aCC-BY 4.0 International license.

Supplementary Tables

Table S1. Evaluation of PADDLE performance. PADDLE was evaluated on the initial dataset and achieved poor prediction performance. The model combines one-hot encodings and secondary structure predictions. However, the secondary structure predictions result in worse performance likely due to increased complexity of input features.

Secondary Structure Included	Pearson r	RMSE
True	0.261	0.336
False	0.338	0.329

Table S2. Evaluation of protein sequence encodings. One-hot encodings outperform other simple protein representations on a held-out test dataset. Neural encodings from pretrained protein language models slightly outperform one-hot encodings on a held-out test dataset. Metrics were averaged across 10 random seeds.

Encoding	Pearson r	RMSE
BLOSUM	0.730	0.692
NLF	0.698	0.722
One-hot	0.742	0.672
ESM1_650M_1	0.740	0.669
ESM1_650M_2	0.732	0.678
ESM1_650M_3	0.734	0.678
ESM1_650M_4	0.739	0.674
ESM1_650M_5	0.728	0.685
ESM2_8M	0.744	0.664
ESM2_35M	0.744	0.664
ESM2_150M	0.725	0.686
ESM2_650M	0.706	0.708
ESM2_3B	0.744	0.668

Table S3. Evaluation of the state-of-the-art model relative to ADhunter. ADhunter outperforms the state-of-the-art AD predictor, TADA, when trained on the initial dataset and evaluated on a held-out test dataset. PADDLE was not included since the untrained model is not available for fair comparison.

Model	Pearson r	RMSE
TADA	0.538	0.995
ADhunter	0.775	0.631

bioRxiv preprint doi: https://doi.org/10.1101/2025.09.12.675635; this version posted October 29, 2025. The copyright holder for this preprint (which was not certified by peer review) is the author/funder, who has granted bioRxiv a license to display the preprint in perpetuity. It is made available under aCC-BY 4.0 International license.

Table S4. TADA ablation study. We evaluated the contribution of each architectural component of TADA by incrementally removing or replacing key elements then training on the initial dataset and evaluating on a held-out test dataset. Metrics were averaged across three random seeds.

Model variant	Accuracy	F1 score
Conv1D-Dropout-Conv1D-Dropout-Attention-BiLSTM-BiLSTM	0.845	0.845
Conv1D-Dropout-Conv1D-Dropout-Attention-BiLSTM	0.845	0.845
Conv1D-Dropout-Conv1D-Dropout-Attention-MaxPooling1D	0.839	0.839
Conv1D-Dropout-Conv1D-Dropout-MaxPooling1D	0.852	0.852
Conv1D-Dropout-MaxPooling1D	0.849	0.849
Conv1D-Dropout-Conv1D-Dropout-BiLSTM-BiLSTM	0.850	0.850
Conv1D-Conv1D-Attention-BiLSTM-BiLSTM	0.846	0.846
Conv1D-Conv1D-BiLSTM-BiLSTM	0.852	0.852
Conv1D-Conv1D-MaxPooling1D	0.866	0.866
Conv1D-MaxPooling1D	0.860	0.860
Attention-MaxPooling1D	0.839	0.838
BiLSTM	0.863	0.863

Table S5. Evaluation of model generalizability. Using spectral clustering analysis, the ensemble model of ADhunter with neural encodings outperforms single models of ADhunter with simple (one-hot) or neural (ESM) encoding at generalizing on the initial dataset. Metrics were averaged across three random seeds.

Model	Pearson r	RMSE
TADA	0.381	1.021
ADhunter_simple	0.416	0.884
ADhunter_neural	0.476	0.856
ADhunter	0.512	0.826

Table S6. ADhunter achieves state-of-the-art performance as a surrogate model for AD activity. ADhunter was compared to the state-of-the-art AD predictor, TADA, when trained and tested on either the harmonized dataset or the Morffy et al. dataset. ADhunter outperformed TADA on both datasets as well as regression and classification prediction tasks. Metrics are averaged across three random seeds.

Prediction Task	Model	Harmonized Dataset	Morffy et al. Dataset
**Regression**	**TADA**	Pearson r = 0.621; RMSE = 0.935	Pearson r = 0.635; RMSE = 0.961
	**ADhunter**	Pearson r = 0.818; RMSE = 0.556	Pearson r = 0.682; RMSE = 0.740
**Classification**	**TADA**	Accuracy = 0.861; F1 score = 0.866	Accuracy = 0.924; F1 score = 0.922
	**ADhunter**	Accuracy = 0.905; F1 score = 0.910	Accuracy = 0.932; F1 score = 0.935

bioRxiv preprint doi: https://doi.org/10.1101/2025.09.12.675635; this version posted October 29, 2025. The copyright holder for this preprint (which was not certified by peer review) is the author/funder, who has granted bioRxiv a license to display the preprint in perpetuity. It is made available under aCC-BY 4.0 International license.

Supplementary Data

Data S1. Initial dataset.
Data S2. Active learning library.
Data S3. Harmonized dataset.
Data S4. Oligonucleotides used in this study.
Data S5. Fluorescence-activated cell sorting metrics.
